# Transporter Regulation in Critical Protective Barriers: Focus on Brain and Placenta

**DOI:** 10.3390/pharmaceutics14071376

**Published:** 2022-06-29

**Authors:** Valerio Taggi, Mario Riera Romo, Micheline Piquette-Miller, Henriette E. Meyer zu Schwabedissen, Sibylle Neuhoff

**Affiliations:** 1 Biopharmacy, Department of Pharmaceutical Sciences, University of Basel, 4056 Basel, Switzerland; valerio.taggi@unibas.ch (V.T.); h.meyerzuschwabedissen@unibas.ch (H.E.M.z.S.); 2 Leslie Dan Faculty of Pharmacy, University of Toronto, Toronto, ON M5S 3M2, Canada; mario.rieraromo@mail.utoronto.ca (M.R.R.); m.piquette.miller@utoronto.ca (M.P.-M.); 3 Certara UK Ltd., Simcyp Division, Sheffield S1 2BJ, UK

**Keywords:** drug transporters, ABC, SLC, regulation, diseases

## Abstract

Drug transporters play an important role in the maintenance of chemical balance and homeostasis in different tissues. In addition to their physiological functions, they are crucial for the absorption, distribution, and elimination of many clinically important drugs, thereby impacting therapeutic efficacy and toxicity. Increasing evidence has demonstrated that infectious, metabolic, inflammatory, and neurodegenerative diseases alter the expression and function of drug transporters. However, the current knowledge on transporter regulation in critical protective barriers, such as the brain and placenta, is still limited and requires more research. For instance, while many studies have examined P-glycoprotein, it is evident that research on the regulation of highly expressed transporters in the blood–brain barrier and blood–placental barrier are lacking. The aim of this review is to summarize the currently available literature in order to better understand transporter regulation in these critical barriers.

## 1. Introduction

**Figure 1:**
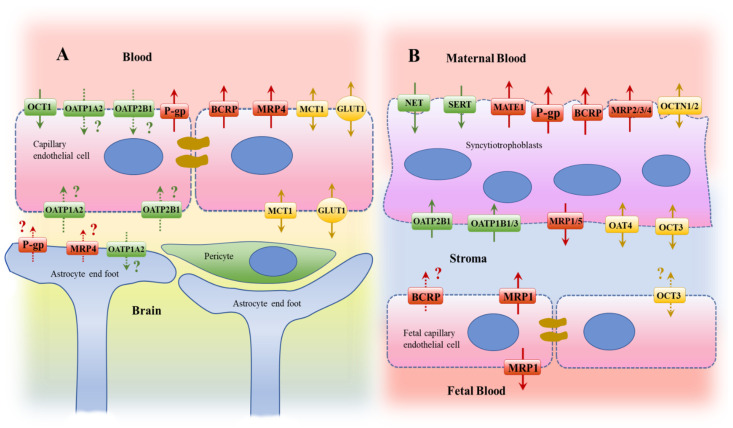
Overview of transporters expressed in the blood–brain barrier (**A**) and the blood–placental barrier (**B**). ABC transporters are represented in red, unidirectional SLC are represented in green and bidirectional SLC are represented in yellow. Arrows indicate the direction of the transport. Dotted arrows with a question mark indicate that the transporter localization requires confirmation.

Critical barriers surrounding the brain and fetus, respectively termed the blood–brain barrier (BBB) and the blood–placental barrier (BPB), form functional barriers between these vulnerable tissues and the systemic circulation, thereby playing a key role in protection against potentially toxic environmental and endogenous compounds. Furthermore, transporter systems within specialized cells of the barriers serve as mechanisms to provide adequate exchange of essential substances, gasses, and wastes. A number of these transport systems are also involved in the exchange of drugs and are highly expressed in the apical and/or basolateral membranes of the BBB and BPB (Figure 1). The proper function and regulation of these transporters is therefore critical to the health of the brain and the developing fetus. Several mechanisms underlying the regulation of drug transporters in the brain and placenta under physiological conditions have been studied. A recent review on behalf of the International Transporter Consortium (ITC) on the regulation of drug transport proteins focused on the key mechanisms and regulatory factors that alter the function of clinically relevant transport proteins involved in drug disposition [1]. The review considers transporters in intestine, liver, kidney, brain, lung, placenta, and other important sites such as tumor tissue. Although studies about dysregulation of transport systems have been reported for several pathological conditions, these could not be addressed in detail in the ITC paper. Our review is a follow-up analysis of the regulation of transporters under pathological conditions focusing only on the protective barriers in brain and placenta. Therefore, this review intends to provide a summary of current knowledge on the regulation of drug transporters in disease and potential underlying mechanisms involved.

## 2. Histological and Transport Characteristics

### 2.1. Brain

As excellently summarized by Daneman and Prat [2], the term blood–brain barrier (BBB) refers to the microvasculature of the central nervous system (CNS), which is a specialized physiological structure composed from various cell types forming continuous nonfenestrated vessels. The luminal site of the vessels is covered with endothelial cells with unique properties that allow them to tightly regulate the transcellular movement. On the abluminal side, the endothelial cells are in close contact with mural cells, astrocytes, and immunological cells, and this interaction is assumed to determine the unique functionality of the BBB. The major function of the multicellular unit at the BBB is to govern the exchange of molecules and cells between the blood and the brain, maintaining the highly selective permeability and regulating uptake and extrusion. The BBB is therefore crucial to preserve CNS homeostasis, protecting it from toxins, pathogens, inflammation, and disease, but at the same time allowing nutrients such as glucose to reach the brain. However, the protective nature of the BBB also provides an effective barrier to drugs, which can be a challenge for brain-targeting therapies (for details please refer to [2,3,4]). It is widely accepted that transporters, or, more precisely, members of the ATP-binding cassette (ABC) transporter family and Solute carrier (SLC) family, play a pivotal role in the functionality of the BBB and are therefore of clinical relevance to drug or endogenous compound disposition (Figure 1).

In this review, we aim to focus on the mechanisms shown to regulate transporters in the endothelial cells of the BBB. The ABC-transporter P-glycoprotein (P-gp), which is encoded by ABCB1/MDR1, is the best-studied transporter in the context of pathologies. In addition to P-gp, multiple other transporters are assumed to contribute to the functionality of the BBB. Ranking transporters for their abundance as detected in isolated human brain microvessels by mass spectrometry suggests higher levels for the glucose transporter 1 (GLUT1/SLC2A1; reported mean value from independent studies ranged from 21.9 to 139.0 pmol/mg total protein) as compared to all other transporters observed in the currently published mass proteomic studies. Of note, there are a limited number of studies that have quantified the abundance of transporters in isolated human brain microvessels and there are inconsistencies in the reported data [5,6,7,8]. However, the breast cancer resistance protein, BCRP (ABCG2; reported study mean values of 2.22–8.14 pmol/mg total protein), and P-gp (reported study mean values of 2.58–6.06 pmol/mg total protein), are consistently detected by all groups and both ABC-transporters are ranked as highly expressed transporters within brain microvessels. Findings on the monocarboxylate transporter 1 (MCT1/SLC16A1; reported study mean values of 1.46 to 5.37 pmol/mg total protein) and the equilibrative nucleoside transporter 1 (ENT1/SLC29A1; reported study mean values of 0.27–0.86 pmol/mg total protein) are also consistent, exhibiting lower amounts than reported for BCRP and P-gp. Other transporters that were detected and quantified by the groups cited above include the organic anion transporter (OAT) 1 (OAT1/SLC22A6), OAT2 (SLC22A7), OAT3 (SLC22A8), OAT7 (SLC22A9), the organic cation transporter (OCT) 1 (OCT1/SLC22A1), OCT3 (SLC22A3), the organic anion transporting polypeptide (OATP) 1A2 (SLCO1A2), OATP2B1 (SLCO2B1), OATP1C1 (SLCO1C1), and the multidrug resistance protein (MRP) 4 (MRP4/ABCC4). However, inconsistencies exist in terms of calculated abundance and peptide data [5,6,7,8,9]. Importantly, the quantitative data from mass spectrometry reported as “pmol transporter/mg total protein” cannot easily be compared between laboratories, as accuracy data of the proteomic methods, which allow one to distinguish between assay and population variability of the measured parameter, or method-specific scaling factors accounting for losses within the tissue preparation, are often not stated or are simply lacking [10].

In addition to abundance, the role of a transporter is also impacted by its localization in the polarized endothelial cells. Determination of ultrastructural localization within endothelial cells of the human BBB allows for prediction of their contribution towards the net transcellular transport of their substrates. Indeed, confocal and electron microscopy analysis has confirmed luminal localization of BCRP and P-gp [11,12], while suggesting luminal and abluminal expression of MCT1 [13] and GLUT1 [14]. For other transporters mentioned above, there is not only inconsistency in the proteomic data sets, but there is also the need to clarify their subcellular localization, in order to specify the direction of their contribution to the transcellular transport at the BBB. This applies for both OATP1A2 and OATP2B1 [15] that have been readily detected in the human brain by mass spectrometry [16,17] and immunohistochemistry [16,18], suggesting, at least for OATP1A2, some expression in glial cells. Similar findings are reported for human MRP4 [19], with rodent data supporting its apical localization in endothelial cells [20,21]. Based on data obtained in rat brain, localization of Oatp1c1 is expected in both the abluminal and luminal membrane of the capillary endothelial cells [22]. However, a comparative study reported much lower immunoreactivity for human OATP1C1 compared to rodents [21]. For Oat3, an abluminal localization is predicted based on its immunological detection in rat brain [21]. Finally, for the organic cation transporter OCT1, there are data suggesting expression at the luminal side as detected in cultured brain microvessel endothelial cells from humans [23]. Taken together, there are multiple transport systems expressed at the BBB with expected relevance for its functionality in terms of protection and exchange.

### 2.2. Placenta

The placenta forms a barrier that serves not only as a physical barrier between maternal and fetal circulation (blood), but also plays a critical role in regulating nutrient and waste exchange between mother and fetus. Thus, the placental barrier protects the developing fetus and later embryo from toxicants and xenobiotics, and it ensures its proper development. The placenta is the largest fetal organ, and its main components include the chorionic villi and the fetal vascular endothelium [24,25]. In detail, shortly after implantation of the blastocyst, the placenta starts to develop, subsequently differentiating into trophoblasts. During the first trimester, a network of blood vessels is established and cellular contact with maternal blood is maximized through formation of chorionic villi. The chorionic villi are covered by a two-layered epithelium of trophoblasts consisting of an outer layer of multinucleated syncytiotrophoblasts underlined by cytotrophoblasts. During gestation, the placenta undergoes continuous vascularization, growth, and development. One of the histological features of placental development is that the layer of cytotrophoblasts becomes discontinuous and that a thinning of the syncytiotrophoblast layer occurs. Permeability and transport properties of placenta, particularly of the syncytiotrophoblast layer, are important in regulating uptake and excretion of nutrients, hormones, and wastes, as well as in the protection from potential harmful xenobiotics [24,25]. Indeed, several key drug transporters are highly expressed in syncytiotrophoblasts and are believed to play a decisive role in controlling fetal exposure to endogenous and exogenous substances. Based on mRNA and quantitative proteomic analysis, the most abundantly expressed placental drug transporters include BCRP, P-gp, MRP1 (ABCC1), OATP2B1 (SLCO2B1), OATP4A1 (SLCO4A1), OCT3, and OAT4 (SLC22A11) [26].

The ABC transporters, BCRP and P-gp, are highly abundant in the syncytiotrophoblasts throughout gestation [27,28,29]. Due to their localization in the apical membrane, BCRP and P-gp are assumed to prevent the entrance of drugs into the placenta and fetal circulation; thus, they are critical to avoid undesirable fetal exposure and subsequent developmental issues. Importantly, BCRP is also expressed in endothelial cells of the fetal capillaries where it serves to extrude substrates from the fetal circulation [30]. While there are discrepancies in literature, the transcript and protein expression of MRP1 has been detected in both syncytiotrophoblasts and fetal capillary endothelial cells [31,32]. Using high resolution confocal microscopy, Granitzer et al. recently demonstrated a preferential expression of MRP1 at the basolateral membrane of syncytiotrophoblasts, while expression was shown in both apical and basolateral membranes of fetal endothelial cells [33].

SLC transporters also play a crucial role in biodistribution and fetal exposure of their substrates as they facilitate the cellular entry of several drugs, thereby impacting either the maternal-to-fetal or fetal-to-maternal net transport, depending on localization and directionality of transport. It is believed that many of the SLC transporters are found within the basolateral membrane of placenta, thereby influencing cellular entry at the fetal side of syncytiotrophoblasts. Indeed, immunofluorescence studies have confirmed localization of OCT3, OATP2B1, and OAT4 within the basolateral membrane of syncytiotrophoblasts [34,35,36,37]. Interestingly, for OATP2B1 and OCT3, there is data also showing their expression in fetal membranes and fetal capillaries, respectively [36,37]. Immunohistochemical and functional analysis have also confirmed preferential localization of the carnitine/organic cation transporter (OCTN2) at the apical membrane of syncytiotrophoblasts [38].

Placental expression data of several other drug transporters has been reported, including for ENTs, concentrative nucleoside transporters (CNTs), and additional members of the MRP family. Their expression, physiological role, and implications in maternal-drug transfer are much less characterized and there are contradictory findings in the literature [31,39]. For example, while ENT1 (SLC29A1), ENT2 (SLC29A2), and CNT2 (SLC28A2) transcripts have been detected in human term placenta, only ENT1/2 expression has been corroborated at protein level [40].

In summary, there are multiple transport systems expressed in the syncytiotrophoblast; however, the current knowledge on transporter-protein abundance and functional implication of alterations in this critical protective barrier is still limited and requires further research.

## 3. Transporter Regulation

### 3.1. Brain

Major neurological diseases, such as multiple sclerosis, stroke, epilepsy, Alzheimer’s disease, and Parkinson’s disease, are linked to structural and functional changes in the BBB [2]. The impact of these neurodegenerative diseases on transporter regulation has been best characterized for the ABC-transporter, P-gp. In the following paragraphs, we will elaborate on the factors and underlying mechanisms involved in transporter regulation in the context of pathological conditions for Alzheimer’s disease, epilepsy, Parkinson’s disease, and, finally, on human immunodeficiency virus (HIV) infection.

#### 3.1.1. Alzheimer’s Disease

Briefly, Alzheimer’s disease (AD) is characterized by the formation of extracellular plaques containing amyloid β (Aβ) and intracellular neurofibrillary tangles containing hyperphosphorylated *tau* protein. Aβ derives from amyloid precursor protein (APP), a single-pass transmembrane protein highly expressed in the brain, which is metabolized through sequential proteolysis by β- and γ-secretase enzymes [41]. According to the amyloid cascade hypothesis, accumulation of Aβ in AD is caused by an imbalance of Aβ production and clearance, while the formation of neurofibrillary tangles is rather considered a subsequent process [42]. Removal of Aβ-peptides from the brain involves multiple mechanisms of the “proteostasis” (reviewed in Hipp et al., 2019 [43]), but also transport. Notably, transport of Aβ across the capillary endothelium of the BBB and via cerebrospinal fluid (CSF) reabsorption into the venous blood accounts for about 50% of the total Aβ-clearance rate [44]. Here, P-gp is assumed to play a role, as it has been shown to transport Aβ-peptides [45,46,47]. The role of P-gp in the clearance of Aβ-peptides is further supported by data obtained in transgenic mice, where microinjected Aβ40 and Aβ42 were cleared at half the rate compared to wildtype animals [48]. In the transcellular transport of Aβ at the BBB, P-gp is assumed to work in concert with the abluminal (brain-facing) LDL receptor-related protein 1 (LRP-1), which removes Aβ from the interstitial fluid (ISF) by endocytosis [49].

**Figure 2:**
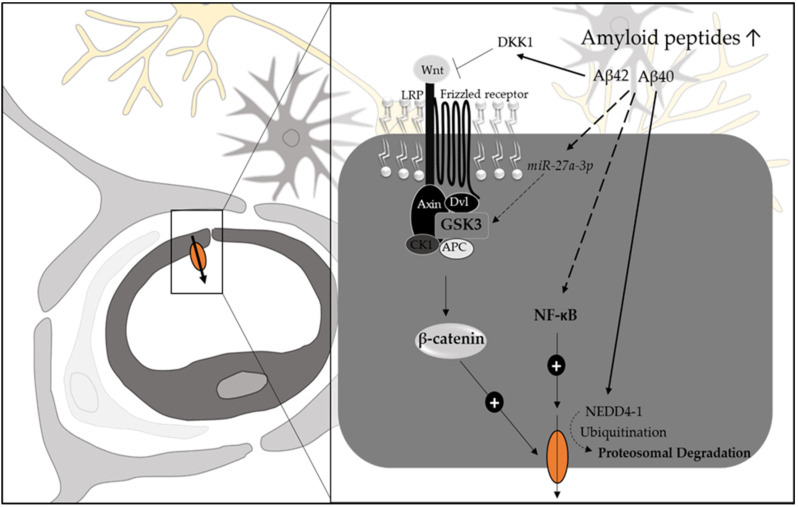
Mechanisms regulating P-gp expression in the blood–brain barrier in Alzheimer’s disease. In AD-affected brain regions, P-gp expression and abundance in the endothelial cells of the blood-brain–barrier is significantly reduced. Various mechanisms have been investigated linking the increased amount of amyloid (A)-peptides Aβ40 and Aβ42 to a reduction in P-gp involving NF-κB, and NEDD4-1 mediated ubiquitination and the Wnt/β-catenin pathway.

Multiple lines of evidence suggest a role of P-gp itself in Aβ-clearance and therefore a link between its modulated expression in the pathogenesis of AD (Figure 2). Indeed, there are various studies reporting reduced expression of P-gp in human AD brains and/or an inverse correlation between P-gp expression and cerebral Aβ-deposition [50,51,52,53,54,55,56,57]. However, targeted proteomic analysis did not reveal changes in P-gp protein abundance (for this and other transporters, including ENT1, OATP2B1, and BCRP) in brain regions affected by AD [17]. The mass spectrometric findings on P-gp are in contrast with the functional (R)-[^11^C]-verapamil PET study results, where AD patients exhibited higher binding potential of this P-gp substrate in most areas of the AD brain and lower extraction ratios compared to age-matched, healthy volunteers [58,59,60]. However, in AD, the binding potential is influenced by genetic variants (C1236T in exon 12, G2677T/A in exon 21, and C3435T in exon 26) of ABCB1 [59]. The frequency of these variants is neither different in AD [61] nor linked to Aβ42 levels in the CSF of AD patients compared to healthy subjects [62].

Importantly, immunohistochemical data show that reduction of P-gp in the BBB is most evident in vessels affected by capillary Aβ-deposits [51,54], suggesting that there is a link or interplay between Aβ-peptides and P-gp expression. Results from several in vitro studies that tested the influence of Aβ40 and/or Aβ42 on P-gp expression support this notion (Figure 2). The Aβ42-mediated reduction of P-gp has been linked to NF-κB-signaling in the murine bEnd.3 cell model [63]. Changes in NF-κB-signaling have also been observed when treating TgSwDI mice with the dual-transporter inhibitor elacridar (BCRP and P-gp inhibitor) to reduce BBB efflux capacity, leading to a further increase in hippocampal Aβ-load and a reduction in P-gp abundance [64]. For Aβ40 treatments, changes in P-gp protein amount were linked to the transporter’s ubiquitination mediated by NEDD4-1, which, due to proteosomal degradation, reduces P-gp surface density [65,66,67]. Enhanced ubiquitination of P-gp has also been observed when comparing capillaries isolated from AD and a normal brain [68]. The third mechanism investigated in the context of amyloid peptides regulating P-gp at the BBB involves the canonical Wnt/β-catenin pathway. Kania et al. showed that treatment of immortalized human brain endothelial cells (hCMEC/D3) with recombinant Aβ40 or Aβ42 significantly reduced abundance of P-gp in these cells. However, only Aβ42 reduces transcription of ABCB1 in vitro [69] and in vivo in mice (treatment 1-day s.c.) [70]. Importantly, the decrease in transcriptional activity was linked to lower amounts of β-catenin protein and an increase in the transcript levels of Dickkopf related protein 1 (DKK1) [69]. Neutralizing the Wnt-pathway inhibitor DKK1 in APPswe/PS1 mice restored β-catenin protein levels, attenuated Aβ pathologies, and increased BBB integrity and P-gp expression [71], further supporting the role of the Wnt/β-catenin signaling in the regulation of P-gp levels in the BBB during Alzheimer‘s disease [72]. In contrast, activation of Wnt signaling increases P-gp expression and barrier function in cultured hCMEC/D3 cells [73]. Finally, miRNAs seem to be also involved in P-gp regulation at the BBB. Indeed, they are significantly reduced in the CSF of AD patients. The miRNA-27a-3p has been shown to be inversely correlated with ABCB1 levels in intestine or leukemia cell lines, respectively [74,75]. In human brain endothelial cells, miR-27a-3p was observed to be a positive regulator of P-gp involving downregulation of the serine/threonine kinase GSK3β in the presence of the miRNA [76]. Downregulation of GSK3β, which is part of the protein complex with axin, adenomatous polyposis coli (APC), and casein kinase I (Ck1), stabilizes β-catenin and its nuclear transcriptional activity [77,78]. Notably, reduction of β-catenin protein levels has also been observed in human AD brain samples [71].

Taken together, there are multiple studies reporting on the regulation of P-gp in AD-affected brains, involving various molecular mechanisms, but whether this is linked to the pathogenesis of AD remains to be determined. Finally, in AD, the amount of *tau* and phosphorylated *tau* protein in CSF increases, while the aggregation-prone Aβ42 but not the soluble Aβ40 is decreased [79,80]. Whether this is linked to P-gp function in the choroid plexus remains unclear, especially when considering that current data on P-gp localization in epithelial cells of the human and rodent choroid plexus are rather inconclusive [81,82,83]. While our current review indicates that available information on transporter regulation in the BBB in AD is focused on P-gp, two excellent reviews list transporter such as ENT1, OATs, OATPs, GLUTs, as well as BCRP, MRP1, ABCG1/4, and ABCA1/7, to be partially involved, either directly or indirectly, in the pathogenesis of AD [84,85]. However, the regulation of these transporters in the AD brain is currently neither quantitatively nor mechanistically evaluated.

#### 3.1.2. Epilepsy

In normal brain, P-gp expression and function appears to be limited to endothelial cells of the BBB, while in epileptic tissue increased (or over-) expression of this efflux transporter is not only observed in endothelial cells of the BBB, but also in astrocytes and/or dysplastic neurons located within affected brain regions [86,87,88,89,90,91,92]. Even though there is a plethora of studies reporting on P-gp overexpression in tissue of epileptic brains, a recent report applying targeted proteomics was not able to confirm upregulation of P-gp or other efflux transporters in epileptic tissue samples [93]. However, there is accumulating evidence that epileptogenic insults induce fundamental structural and functional changes in the BBB, resulting in a loss of its integrity with increased permeability [94]. As stated by Löscher and Friedman in 2020, upregulation of efflux transporters (P-gp, MRP1, MRP2) in brain capillary endothelial cells and/or perivascular glia in the context of reduced BBB integrity in response to seizures can be considered a “second line of defense mechanism” [95]. In line with this notion are clinical and preclinical findings linking seizures or epileptic activity to an induction of P-gp expression and function. Moreover, pharmaco-resistance to antiepileptic drugs (AEDs) has also been linked to an increased expression and function of efflux transporters in affected brain areas [96]. Notably, in the context of refractory epilepsy, the so-called transporter hypothesis is one of the six proposed potential mechanisms contributing to AED resistance [97].

**Figure 3:**
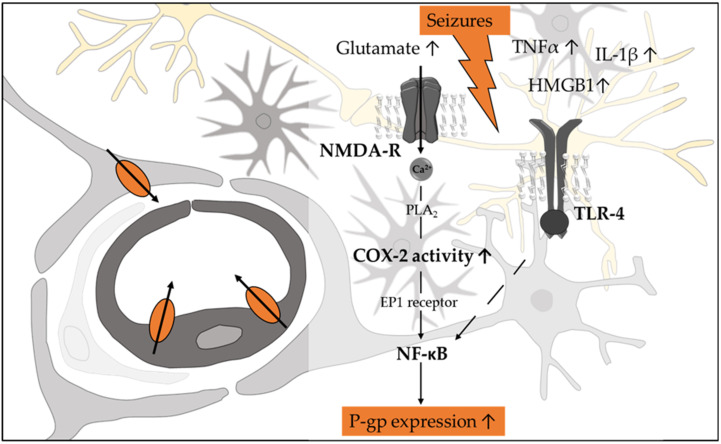
Schematic on the mechanisms assumed to be involved in seizure/epilepsy-induced upregulation of P-gp expression in affected brain tissue. Seizures increase the amount of glutamate-activating N-methyl-D-aspartate receptor (NMD-R) and thereby the calcium content. Activated phospholipase A*2*. (PLA2) cleaves phospholipids generating the cyclooxygenase 2 (COX-2) substrate arachidonic acid, which is catalyzed forming prostaglandin E2 (PGE2). PGE2 is assumed to activate the PGE2 receptor EP1, which modulates P-gp expression involving NF-κB. This transcription factor is also modulated by involving the Toll-like receptor 4 (TLR-4) and the inflammatory mediators: interleukin (IL)-1β, tumor necrosis factor (TNF)-α, and high-mobility group box-1 (HMGB1).

One molecular mechanism intensively studied in the context of seizure-induced P-gp expression involves glutamate, the N-methyl-D-aspartate (NMDA) receptor, cytosolic phospholipase A2 (cPLA2), and the cyclooxygenase-2 (COX-2) (Figure 3) [95,98]. High levels of extracellular glutamate [98] are assumed to contribute to various neurodegenerative diseases including epilepsy [99,100]. In rat BMECs, glutamate was shown to increase P-gp expression in an NMDA receptor-dependent manner [101]. NMDA receptor activation results in calcium influx, which activates cPLA2. In line with a role of cPLA2 in the endothelial upregulation of P-gp, glutamate was found to increase cPLA2 activity in vitro, and the inhibition of this enzyme in vivo reduced seizure-induced upregulation of P-gp [102]. cPLA2 cleaves phospholipids generating the COX-2 substrate arachidonic acid, which is catalyzed forming prostaglandin E2 (PGE2). The link between P-gp expression and COX-2 activity is clearly supported by findings in animal models, where seizure-induced upregulation of P-gp was attenuated by COX inhibition [103,104]. Profiling mRNA expression in peripheral blood samples of patients characterized as valproate responders and valproate nonresponders revealed COX-2 (PTSG2) as the most significantly downregulated gene, which was translated into significantly lower PGE2 levels in plasma of the responders [105]. Finally, antagonism of the prostaglandin E2 receptor EP1 abolished seizure-induced P-gp upregulation in endothelial cells of rats [106]. Transcriptional regulation of P-gp via this pathway is assumed to involve NF-κB, which plays a central role in the pathogenesis of neuroinflammation in the context of epilepsy [107]. Various neuroinflammatory cascades become initiated in response to seizure-induced neuronal injury with an increased production/release of proinflammatory mediators including interleukin (IL)-1β, IL-6, tumor necrosis factor (TNF)-α, and high-mobility group box-1 (HMGB1) [108]. This led to the linking of seizure/epilepsy as a stress stimulus to the NF-κB signaling pathways [109]. The impact of the abovementioned cytokines (IL-1β, TNF-α, IL-6) on P-gp expression in endothelial cells of the BBB are rather inconsistent [110,111,112,113]. However, there are recent data linking the Toll-like receptor (TLR)-4 to higher levels of IL-1β, TNF-α, and NF-κB in brain and P-gp overexpression in drug-resistant epileptic rats. Indeed, in TLR-4-deficient drug-resistant epileptic rats, the levels of the cytokines and NF-κB were significantly reduced, and this was accompanied by a reduction in P-gp abundance and expression in the hippocampus and amygdala [114]. A link between TLR-4, NF-κB, and P-gp expression was also observed, determining the function of miR-542-3p in a rat epilepsy model, where this particular miRNA was shown to modify TLR-4 expression [115]. In addition to the cytokines, seizure-induced tissue damage has been linked to enhanced release of HMGB1, which is assumed to play a role in inflammation and BBB disruption [116,117]. In vivo and in vitro data correlate increased levels of HMGB1 to seizure-induced upregulation of P-gp [118]. The mechanism involves TLR-4, the receptor for advanced glycation end products (RAGE) and activation of NF-κB in mouse microvascular endothelial cells [119]. Similar results linking HMGB1 directly to P-gp overexpression were described in leukemic cell models [120,121].

As recently summarized by Ghafouri-Fard et al., there are several miRNAs that are differentially expressed in animal models of epilepsy and in the brains of patients with epilepsy [122]. Some of them have been investigated for their role in P-gp upregulation. Among the miRNAs downregulated in the context of epilepsy is miR-289, which has been shown to reduce expression of P-gp in drug-resistant hBMEC [123]. Among the miRNAs upregulated in epilepsy is miR-146a, which is considered a prognostic biomarker predicting development of drug resistance [124]. Antagonism of miR-146a has been shown to reduce inflammatory cytokines and expression levels of P-gp in the brain tissue of epileptic rats [125]. Finally, the miR-27a-3p was observed to be reduced in the serum of epileptic mice and humans [126], while increased levels of miR-27a-3p were demonstrated in the hippocampus of epileptic rats [127]. This particular miRNA has been shown to upregulate P-gp expression and function in endothelial cells as described in the paragraph about Alzheimer’s disease [76].

Taken together, there is some evidence linking epilepsy to an upregulation of P-gp with evidence from rodent models indicating that overexpression may contribute to drug resistance. It appears noteworthy at this point that there are rodent in vivo and in vitro data that antiepileptic drugs such as phenobarbital, phenytoin, carbamazepine, and valproic acid increase P-gp expression and function in the brain [128,129]. Nevertheless, we lack studies investigating their effect on these transporters in humans. Finally, writing this review on both the BBB and the BPB, we want to mention that epilepsy and schizophrenia are the most common neurological conditions observed in women at childbearing age. As these women need to be maintained on therapeutic regimens throughout pregnancy, we want to highlight that there are data suggesting potential regulation of placental transporters by antiepileptic drugs including phenytoin, valproic acid, carbamazepine, levetiracetam, and lamotrigine. These drugs have been reported to modulate mRNA expression and/or protein levels of folate, fatty acid, and/or amino acid transporters, as well as BCRP and OATPs, in human placental cell lines and explants [130,131,132]. In terms of underlying regulatory mechanisms, the majority of AEDs were found to modulate transporter expression through activation of nuclear transcription factors such as PXR, PPARα, and PPARγ [131]. In the case of valproic acid, evidence of drug-induced histone acetylation was also seen, suggesting further regulation through epigenetic regulatory pathways [130,132].

#### 3.1.3. Parkinson’s Disease

Parkinson’s disease (PD) is one of the neurodegenerative conditions where expression of P-gp has been investigated as a representative of the efflux transporter family contributing to barrier function. In general, PD is characterized by the progressive loss of dopaminergic neurons within the substantia nigra and the intracytoplasmic accumulation of abnormal aggregates of misfolded α-synuclein protein (Lewy bodies) [133,134]. The progressive depletion of dopaminergic neurons in the nigrostriatal pathway will result in the typical parkinsonian motor symptoms, but also in nonmotor symptoms as excellently summarized by Kalia and Lang in 2015 [135]. However, the authors emphasize that the so-called Lewys pathology is not limited to the substantia nigra.

One feature of the PD brain is the structural and functional alteration in the BBB’s integrity. Indeed, vascular degeneration has been shown histologically [136] and functionally [137]. One of the factors that may contribute to a change in functionality is the expression of the efflux transporter P-gp. So far, data on P-gp expression in PD is limited and is mainly based on animal studies. In detail, in a murine PD model of 1-methyl-4-phenyl-1,2,3,6-tetrahydropyridine (MPTP)-induced dopaminergic degeneration, the authors observed an increased level of P-gp substrate bromocriptine in the brain, suggesting either disruption of BBB or alteration of active efflux of the drug. However, no alteration in BBB integrity was observed when assessed by in situ brain perfusion with inulin or sucrose, respectively. Importantly, similar results were obtained for the BBB functionality tested with digoxin (P-gp substrate) and prazosin (BCRP substrate). For both compounds, no difference in brain/plasma ratios was observed, even though the authors detected a slight but significant increase (1.43-fold) in P-gp mRNA expression in isolated brain microvessels, while the Bcrp mRNA was reduced by 30% [138]. In another PD mouse model, Carvey et al. reported an increased microvascular leakage using FITC-labeled albumin in murine striatal brain areas injected with 6-hydroxydopamine (6-OHDA). In addition, the authors also observed an extended immunofluorescent signal for P-gp, with immunoreactivity in additional cell types in affected areas [139]. However, Kim et al. recently reported a significant reduction in the mRNA and protein expression of endothelial P-gp in both the 6-OHDA-induced (reduction by 50%) and the sporadic α-synucleinopathy (reduction by 80%) mouse models of PD [140]. Finally, in rats, Huang et al. showed no change of P-gp protein in the cortex after injection of 6-hydroxydopamine into the left medial forebrain bundle [141]. However, the authors reported significant downregulation of P-gp by treatment of the disease model rats with levodopa and linked that to an advantage for the treatment with P-gp substrates. Taken together, the data from animal studies in terms of P-gp expression in PD disease models are rather inconclusive but do support a change in vascular morphology with changes in integrity and permeability in association with the progression of the disease. Similar data are reported for the human PD brain. In detail, PD-affected brain areas exhibit pronounced alterations in the capillary network with a lower number, shorter length, and larger diameter of brain capillaries with significantly reduced branching compared to age-matched, healthy controls [136]. In addition to the morphological changes, loss in integrity of the human BBB with increased permeability is assumed, as supported by findings of Gray and Woulfe, who histologically assessed serum protein, iron, and erythrocytes in striata of PD patients, revealing enhanced extravasation in comparison to control samples [142]. In accordance with a reduced tightness are findings in postmortem brains of patients dying with PD, showing significantly reduced abundance of tight junction proteins such as Zona occludens-1 (ZO-1) and occludin [143]. However, as excellently summarized by Cabezas et al., it is assumed that there are multiple mechanisms and various cell types involved in the phenomenon of a lost or reduced BBB integrity [144]. One cell type noteworthy in this context is the astrocyte type, which is assumed to undergo phenotypical changes during the development and progression of PD, resulting in a reactive gliosis, which has a detrimental effect on the barrier function (summarized in [144]). In addition, vascular degeneration has been linked to thickening and collagen accumulation in the capillary basement membrane [145].

In terms of estimating the impact of PD on the expression and activity of P-gp in humans, direct measurements are rather limited [146]. Therefore, we are going to focus on PET studies reporting on [^11^C]-verapamil. In 2005, Kortekaas et al. found enhanced signals (18%) in the midbrain region of five PD patients compared to five healthy controls measured with [^11^C]-verapamil, thereby suggesting a reduction of P-gp function associated to the disease [147]. However, in patients with early-stage PD using [^11^C]-(R)-verapamil as the PET tracer, the same group did not observe these changes in the midbrain even if they reported a larger variation in the volume of distribution of verapamil in PD patients compared to healthy controls [148]. Finally, in 2008, Bartels et al. reported [^11^C]-verapamil PET data of an extended study population showing enhanced [^11^C]-verapamil uptake in frontal white matter regions of advanced PD patients compared to controls, suggesting a regional downregulation of P-gp function [149]. From these functional studies, one tends to conclude that P-gp function is reduced at higher disease stages; however, we have to keep in mind the changes in BBB integrity, which certainly may also influence brain entry of a P-gp substrate.

#### 3.1.4. Human Immunodeficiency Virus Infection

For the BBB, we have so far focused on neurodegenerative diseases and their impact on the regulation of drug transporter expression and function. However, these diseases are commonly linked to neuroinflammation, with respective changes in cytokine profiles and various regulatory pathways. Neuroinflammation, as an underlying mechanism of changes in transporters expression and function in the BBB, is also assumed for infectious diseases. One major disease which should be considered to be of high interest would be the Human Immunodeficiency Virus (HIV) infection. Here, the CNS is considered a sanctuary site, an anatomical compartment where HIV replication can take place in the presence of Highly Active Antiretroviral Therapy (HAART) due to poor drug exposure [150]. The BBB, and certainly the function of the highly expressed efflux transporters, P-gp and BCRP, which transport a variety of antiretroviral drugs [151], should be considered to be of major importance in this context.

For P-gp, there are data showing higher amounts of this efflux transporter in patients with HIV-encephalitis [152] with significantly enhanced immunolabelling of astroglia cells. Importantly, no significant difference in amount, localization, or immunolabelling of astroglial cells was observed for seronegative controls or patients with HIV without encephalitis [152]. Considering those data, it has been investigated whether shed viral proteins such as the glycoprotein-120 (gp120) or the transactivator of transcription (Tat) impact expression of transporters in preclinical BBB models. Briefly, in an HIV-1 transgenic rat model which is characterized by circulating levels of gp120 and Tat, the authors reported downregulation of the mRNA of most of the assessed efflux transporters (Abcb1, Abcb1b, Abcg2, Abcc1 and Abcc4) at least in older rats [153]. However, testing the impact of gp120 or Tat in astrocyte or endothelial models of rodent or human origin revealed rather conflicting data [154,155,156,157,158]. In human CD4+ T-cells, exposure to an HIV pseudotype significantly upregulated P-gp, BCRP, and MRP1, most likely involving inflammatory cytokines [159]. Release of TNF-α, IL-6, or IL-1β upon treatment with gp120 has been shown in vitro for astrocytes [157,160,161] and in vivo [162,163,164,165,166]. Notably, as summarized by Persidsky and Gendelman, HIV-1-infected macrophages and microglia produce multiple factors including glutamate, inflammatory cytokines, and chemokines, that may alter the BBB function [167]. Especially for glutamate and inflammatory cytokines, an influence on P-gp expression in astrocytes and endothelial cells may be expected in a manner similar to how it was elaborated on in the above paragraph on epilepsy. However, whether this impacts functionality of the BBB especially for antiretroviral drugs remains to be systematically investigated.

### 3.2. Placenta

Throughout pregnancy, the placenta undergoes morphological transformations along with gestational-dependent changes in transporter expression. Although there are discrepancies in earlier reports, recent proteomic analysis has shown a gestational decline in protein expression of BCRP and P-gp, decreasing nearly two-fold from the first trimester to term. On the other hand, levels of OAT4 and OCT3 were found to increase from the first or second trimester to term [26]. Various intrinsic and extrinsic factors have also been shown to regulate transporters in placenta (Table 1, Table 2 and Table 3).

Progesterone and estradiol, steroid hormones which physiologically fluctuate during pregnancy, have been shown to increase placental expression of BCRP and P-gp, while maternal administration of glucocorticoids decrease levels of OATP2B1 and BCRP [184,185,186]. Changes in oxygen tension within the placental circulation also occur throughout pregnancy, and this can be affected by maternal conditions such as preeclampsia. Studies in cultured placental explants and BeWo cells have shown that hypoxia alters expression of a number of transporters including BCRP, P-gp, OATP2B1, and several MRPs [180,187]. Hypoxic conditions were also found to impact expression of regulatory nuclear factors involved in transporter regulation, including the Aryl hydrocarbon receptor (AHR), Nuclear factor erythroid 2-related factor 2 (NRF2), and Retinoid X receptor alpha (RXR-α) [187]. Endogenous signaling proteins affecting growth or angiogenesis, such as the epidermal growth factor, insulin growth factor, and the soluble fms-like tyrosine kinase-1 (sFlt-1), have been shown to regulate BCRP [28,174,181,188].

In addition to these physiological factors, several maternal diseases such as preeclampsia, gestational diabetes, and bacterial or viral infection have shown an overall dysregulation of drug transporters [169,174,176,177] (Table 1, Table 2 and Table 3).

**Table 2:** Summary of ABCG2/BCRP alterations in the placenta in different physiological/pathological conditions.

Condition/Factor	Effect			Reference
	mRNA	Protein	Other	
**Based on Clinical Data**				
Gestational Age	↔	↓ ~2-fold from T1 to T2 (variable)	-	[27]
Gestational Age	-	↓ 55%	-	[26]
Chorio (Preterm)	↑ 1.72-fold	Ns (↑ trend)	mRNA + corr with Chorio degree	[171]
Chorio (Term)	↓ 34%	↔		[185]
Chorio (Preterm)	↑	Ns (↑ trend)	mRNA + corr to IL-8	[170]
Preeclampsia	↓ 40–60%	↓ 45%	-	[174]
Preeclampsia—HELLP	↓	↓	No corr with maternal or umbilical cord TBA levels	[189]
IUFGR	↓	-	mRNA + corr with ABCB1 mRNA	[172]
Diabetes	↔	↔	↑ + corr with ↑ HbA1c plasma)	[176]
HIV	↓ 38%	↔	-	[177]
Hepatitis	↑1.8 fold (trend, *p* = 011)	↑ 2.3-fold	-	[178]
Estrogen	-	-	+ corr with maternal estradiol at mid-gest not term	[185]
Nuclear factors	-	-	mRNA + corr with AHR and NRF2 mRNA	[190]
Prenatal Dexamethasone	-	↓ 0.5-fold	GR activation	[191]
**Based on Placental Explants**				
LPS	↓ (T1)	↓ (T1)	-	[179]
Poly I:C	↔ (Term)	↔ (Term)	-	[179]
Hypoxia	↔/↓	↔ (Term)	↓ AHR, NRF2, RXRα, ↑PPAR-Υ mRNA	[180]
Th17 cytokines: IL-17/22/23 combo	↓	↔	-	[192]
Estradiol	↓ (Term)	-	-	[193]
cART treatment	↑ 1.6- to 1.9-fold	-	-	[177]
**Based on Primary Trophoblasts**				
TNF-a	↓ >40%	↓ 50%	↓ function	[181]
IL-1b	↓ >40%	↓ 50%	-	[181]
Epidermal growth factor	↑ >120%	↑ ~90%	↑ function	[181]
Insulin-like growth factor	↑ ~70%	↑ ~50%		[181]
Estradiol	↑ >50%	↑ >50%		[181]
Prostaglandin E2	↑ 1.5-fold	↑ 1.6-fold	Via EP receptors in PHT	[183]

**Chorio**: Chorioamnionitis. **Ns**: nonsignificant. corr: correlated. **HELLP**: Hemolysis elevated liver enzymes and low platelets syndrome. **TBA**: Total bile acids. **IUFGR**: Intrauterine Fetal Growth Restriction. **HbA1c**: Hemoglobin A1c (glycated hemoglobin). **AHR**: Aryl-Hydrocarbon Receptor. **NRF2**: Nuclear factor-erythroid factor 2-related factor 2. **GR**: Glucocorticoid Receptor. **cART**: Combination Antiretroviral Treatment. **PHT**: Primary Human Trophoblasts. **↓**, **↑** and **↔** stand for decrease, increase and no change, respectively, relative to control or stated condition.

Many of these diseases or conditions are associated with elevated immune responses and induction of TNF-α, IL-1β, IL-6, and IL-17. Involvement of inflammation in regulatory control of placental transporters is highly plausible as treatment of primary cultures of placental explants or trophoblast with proinflammatory cytokines have been reported to alter expression of several transporters [181,188]. Drug therapy, such as combination antiretroviral therapy in HIV, could also play a role in transporter dysregulation [177]. Dysregulation of placental transporters with potential consequences for the transcellular transport, and therefore fetal exposure, is particularly concerning, as many of these women require drug therapy throughout pregnancy [194,195].

**Table 3:** Summary of alterations for key SLC in the placenta in different physiological/pathological conditions.

Condition/Factor	Effect			Reference
	mRNA	Protein	Other	
**SLCO2B1/OATP2B1**				
Based on Clinical Data				
Gestational Age	-	↓ 32% between T1 and T2		[26]
Chorio (Preterm)	↓ 57%	↓ 68% (trend, *p* = 0.05)	mRNA (-) corr with IL-1β and IL-6	[169]
Chorio (Term)	-	↓ 49%	-	[169]
HIV	↓ 85–99%	-	-	[177]
Preeclampsia	↑ 1.8-fold	↑		[174]
Prenatal Dexamethasone	-	↓ 0.75 to 0.5-fold	GR activation	[191]
Based on Placental Explants				
Th17 cytokines:				
IL-17, IL-22, IL-23	↓	-	-	[192]
IL-23, and combo	-	↓	-	[192]
Hypoxia	↓ 75%	-	-	[180]
**SLC22A11/OAT4**				
Based on Clinical Data				
Gestational Age	-	↑ 1.6-fold from T2 to Term	-	[26]
Preeclampsia	↓ 50–70%	-	-	[174]
HIV	↓ 85–99%	-	-	[177]
Based on Placental Explants				
Th17 cytokines: IL-23	↓	-	-	[192]
Hypoxia	↓ 25%	-	-	[180]
cART treatment	↓	-	-	[177]
**SLC22A3/OCT3**				
Based on Clinical Data				
Gestational Age	-	↑ 2-fold from T1 to Term	-	[26]
Preeclampsia	↓ 50–70%	↑	-	[174]
HIV	↓ 85–99%	↓ 50%	-	[177]
Based on Placental Explants				
Th17 cytokines: IL-17/22/23 or combo	↔	↔	-	[192]

**T1**: First trimester. **T2**: Second trimester. **Chorio**: Chorioamnionitis. **GR**: Glucocorticoid Receptor. **cART**: Combination Antiretroviral Treatment. **Corr**: correlated. **↓**, **↑** and **↔** stand for decrease, increase and no change, respectively, relative to control or stated condition.

#### 3.2.1. Preeclampsia

Preeclampsia is a highly prevalent worldwide disorder of pregnancy which is characterized by new-onset maternal hypertension and proteinuria along with other biochemical signs of kidney or organ damage. It remains as one of the most common causes of maternal and fetal morbidity [196]. Its etiology is poorly understood but the most accepted theories, according to the current evidence, describe the pathogenesis of the disease as a defective spiral artery remodeling that leads to placental ischemia, imbalance between antiangiogenic and proangiogenic factors, immune activation, and endothelial dysfunction (reviewed in [197,198]). The resulting increase in systemic levels of proinflammatory cytokines as well as placental hypoxia have each been shown to impact transporter regulation [181,187,192]. Indeed, changes in expression of numerous transporters along with alterations in transcript levels of cytokines and growth factors were seen in placenta obtained from women with preeclampsia [170,174]. These samples compared to gestation age-matched controls, displayed significantly decreased transcript levels of BCRP, MRP1, OCT3, OAT4, and ENT2, along with increased levels of OATP2B1 [174].

In line with this, Dunk et al. (2018) reported a significant downregulation of P-gp in villous and extravillous trophoblast from preeclamptic patients and further conducted a series of siRNA knockdown and functional experiments in primary and continuous cultures of cytotrophoblasts and trophoblasts. The authors demonstrated a role of P-gp in trophoblast migration, invasion, and differentiation, suggesting that P-gp downregulation is involved in the pathogenesis of the disease, rather than a consequence [175].

In the same way, placental expression of BCRP and its association with preeclampsia has been investigated. A downregulation of BCRP was seen in placenta obtained from preeclamptic women complicated with hemolysis-elevated liver enzymes and low platelets (HELLP) syndrome as compared to healthy or preeclamptic patients [188]. A subsequent study by Afrouzian et al. demonstrated altered patterns of BCRP expression in preeclamptic placenta. Although BCRP mRNA levels were not altered in placental tissue, increased syncytial knotting was seen in preeclamptic placenta along with increased BCRP staining within the apical membrane of these syncytial knots. Preeclampsia was also associated with increased mRNA and protein expression of MRP1 [199]. Permutations in the expression and release of angiogenic factors are seen in preeclampsia and therefore could play a role in transporter dysregulation. Serum levels of the antiangiogenic factor, sFlt-1 (also known as the soluble VEGF receptor), are elevated during preeclampsia and are thought to be involved in the pathogenic mechanisms of the disease [200]. Binding of sFlt-1 to the vascular endothelial growth factor (VEGF) and placental growth factor (PLGF) blocks activation of the angiogenic vascular endothelial growth factor receptor (VEGFR). As compared to gestational age matched controls, a two-and-a-half-fold increase in placental expression of sFlt-1, along with pronounced decrease in BCRP expression, were seen in placentas obtained from preeclamptic patients [174]. Subsequent in vitro experiments in BeWo cells demonstrated that treatment with sFlt-1 significantly decreased the mRNA and protein levels of BCRP, which is an effect that could be rescued by VEGF. The reduced expression of BCRP was corroborated at a functional level, with sFlt-1 treatments causing decreased efflux and increased intracellular accumulation of the BCRP substrate, topotecan [174]. Altogether, these results demonstrate a reduction in the expression and function of BCRP in preeclamptic placenta and suggest that these effects could be mediated through a combination of hypoxia-related factors, immune activation, and angiogenesis.

#### 3.2.2. Metabolic Disorders

One of the most common complications during pregnancy is gestational diabetes mellitus (GDM), which is characterized by a persistent hyperglycemia that develops during pregnancy and resolves after birth [201]. Numerous metabolic and hormonal changes associated with GDM have the potential to interfere with transporter homeostasis. Preclinical studies using a rodent model of GDM revealed increased expression of P-gp in placenta of pregnant diabetic rats, along with decreased maternal-fetal transfer of the P-gp substrate, lopinavir [202]. Increased hepatic expression of PXR was seen in this model, suggesting PXR-mediated induction of P-gp. This has also been examined clinically, using human samples obtained from GDM pregnancies. In line with the rodent data, Anger et al. demonstrated significantly increased mRNA expression of *MDR1* (P-gp) in placenta obtained from insulin-managed GDM patients as compared to gestation-matched controls. Although significant changes in the expression of other ABC transporters such as BCRP were not seen between groups, a significant positive correlation between BCRP expression (mRNA and protein) and levels of hA1c (glycated hemoglobin, a biomarker of glycemic control) was observed in diabetic patients [176]. Kozlowska-Rup et al. also reported GDM-mediated changes in placental expression but not in localization for P-gp and MRP1 [203]. Interestingly, they found a decrease rather than increase in protein expression of P-gp and MRP1 in GDM placenta.

In addition to regulation of the drug transporters in placenta, there are data linking gestational diabetes to aberrant adenosine transport in placental vascular cells. Indeed, there are preclinical data indicating that nucleoside transporter expression is affected in this metabolic condition. In this context, different groups have evaluated the role of ENT1 and ENT2. In human umbilical vein endothelial cells (HUVECs) exposed to high levels of D-glucose, a reduced adenosine transport was linked to decreased mRNA expression of ENT1/2 [204]. Similar findings were obtained in endothelial cells isolated from umbilical cords of GDM pregnancies [205]. Importantly, in both studies, insulin was able to restore the hyperglycemia-mediated reduction in adenosine transport through an increase in the expression of ENT2 and ENT1, respectively. This was corroborated in patient-derived placental microvascular endothelium cells (hPMEC), showing that cells from GDM had decreased ENT1/2 expression and activity, as well as decreased mRNA expression of insulin receptors IR-A and IR-B [206]. The authors demonstrated that insulin treatments normalized p42/44(mapk)/Akt ratios, increased expression of IR-A/IR-B and ENT2, and restored adenosine transport, thereby reversing the metabolic aberrant phenotype of hPMEC cells. Whether changes in adenosine transport and placenta exposure affects expression of other transport systems remains unclear.

Other factors associated with metabolic disease, such as obesity, may potentially impact transporter regulation; however, there are relatively few studies in the literature. Wang et al. [173] recently reported that P-gp expression was significantly decreased in placenta obtained from obese women as compared to normal-weight women. Likewise, their preclinical studies in a high-fat dietary model of obesity demonstrated a downregulation of P-gp in placenta of obese mice, which was accompanied by increased plasma levels and mRNA placental expression of the proinflammatory cytokines, TNF-α and IL-1β. Increased transplacental transport of digoxin along with increased fetal exposure was associated with the observed changes in P-gp expression.

In the same way, the persistent increase of maternal blood cholesterol in pregnancy, a condition known as maternal supraphysiological hypercholesterolemia (MSPH), has been shown to impact placental ABC transporter expression. The research of Fuenzalida et al. revealed that this condition can cause an increase in the expression of cholesterol efflux regulatory protein or CERP (ABCA1) in placental tissue and primary human trophoblasts from women with MSPH, whereas the expression of ABCG1 (ABC8) was decreased [207]. These results indicate that hypercholesterolemia can affect the cholesterol trafficking itself, increasing the risk of cholesterol accumulation in the fetus, the occurrence of metabolic complications, and possibly inducing fetal exposure to other drugs.

#### 3.2.3. Human Immunodeficiency Virus (HIV) and Hepatitis Infections

The impact of HIV on expression of transporters has also been investigated in the context of pregnancy. Several preclinical and clinical studies have revealed that changes in placental transporters that have been reported in HIV can be related to the pathogenesis of the disease itself or can be induced by pharmacological treatment of the disease. Immune dysregulation is a hallmark of chronic HIV infection. Studies in HIV-1 transgenic (HIV-Tg) rats found that, while basal expression of transporters in placenta were similar to that of wild-types, exposure to low levels of endotoxin resulted in augmented inflammatory responses and amplified dysregulation of transporters in the HIV-Tg rats [208]. In the context of drug-regulated changes, there are experimental data from healthy, pregnant mice that suggest that administration of combination antiretroviral therapy (cART) (consisting of abacavir, lamivudine, atazanavir, and ritonavir) results in a significant downregulation of Bcrp, Mrp1, and Ent1 in placenta [209]. Clinical studies using placental samples obtained from therapeutically managed HIV(+) patients observed increases in mRNA and protein expression of P-gp as compared to uninfected controls. These changes were similar in patients managed with either zidovudine alone or in combination with nelfinavir and/or lamivudine [210]. Likewise, a recent study by Kojovic et al. [177] also reported upregulation of P-gp in placentas isolated from cART-managed HIV(+) women, along with significant downregulation of BCRP, MRP1, MRP2, MRP4, OATP2B1, OCT3, and OAT4 [177]. The study also confirmed involvement of drug therapy in transporter dysregulation as treatments of primary cultured human placental explants with several clinically important ARTs was associated with P-gp induction. 

Although the effect of hepatitis infection on transporter regulation during pregnancy has not been extensively explored, a few studies suggest that hepatitis may have an impact. The use of PCR array screening in pregnant woman with Hepatitis C Virus (HCV) has revealed the increased expression of P-gp and BCRP in comparison to uninfected/healthy controls, while the nucleoside transporters ENT1 and ENT2 remained unchanged. This was corroborated with qRT-PCR and Western blot experiments [178].

## 4. Summary Remarks and Future Directions

Transport proteins play a critical role in the maintenance of homeostasis in different organs. While there is clear evidence that transporters play a pivotal role in controlling access and accumulation of substrates in brain and placenta, there is increasing preclinical and clinical evidence demonstrating disease-mediated regulation within these protective barriers. Maintaining the integrity and functionality of these membrane barriers are essential to the proper functioning of the brain and placenta. More importantly, a large number of drugs used for therapeutic management are substrates of these transporters; hence, modulation of their expression and function may have an impact on their distribution, efficacy, and toxicity.

As we have discussed throughout the review, changes in expression for certain transporters can not only be considered a consequence of a pathological condition but may also be involved in the pathogenesis or even progression of certain diseases. This is especially true for Alzheimer’s Disease in the brain. In neurological diseases, P-gp is the most-studied BBB transporter. Since P-gp is assumed to be involved in the clearance of Aβ peptides, multiple studies suggest a role of this efflux transporter in the development of AD. Various molecular mechanisms and regulatory pathways are associated with downregulation of P-gp, such as NF-κB activation, P-gp ubiquitination, and suppression of the Wnt/β-catenin pathway. Nevertheless, whether these findings can link decreased expression and activity of P-gp to the pathogenesis of AD remains to be determined. Contrarily, P-gp levels are increased in epilepsy. One mechanism that might be involved includes glutamate-mediated activation of the NMDA receptor and COX-2 that together with TLR-4 lead to NF-κB activation, which plays a key role in the development of neuroinflammation. Likewise, neuroinflammation caused by HIV infection may alter BBB functionality and lead to P-gp upregulation. On the other hand, experimental data regarding P-gp are rather contrasting and inconclusive in PD, but highlight changes in vascular morphology, integrity, and permeability of the BBB.

Finally, for most of the diseases mentioned in this review, there is a lack of clinical data concerning transporters’ regulation at the BBB, since data are mainly obtained from in vitro and animal studies. Although this information is relevant, clinical in vivo studies are needed to confirm these findings. In addition, reported data is often conflicting and inconclusive. Therefore, deeper investigations into transporter regulation, with a particular focus on human studies, is needed to optimize therapeutic dosing regimens in pathological conditions. For brain-targeted therapies, the poor penetration of drugs into the CNS is one of the biggest issues limiting the efficacy of current treatments, although efflux at the BBB is crucial to the safety profile for many other drugs. For example, ivermectin, loperamide, non-sedating antihistamines, and antimuscarinics would likely cause more CNS side effects and would have less applications without their efflux at the BBB. This pharmaco-resistance phenomenon involves either reduced uptake or increased efflux of drugs to and from the brain tissue; consequently, alterations in the levels of drugs transporters expressed at the BBB have a crucial implication in therapeutic efficacy or failure. A better understanding on the regulation of transporters at this localization will help to improve the current therapeutic interventions for neurological disorders.

Likewise, the regulatory factors contributing to modulation of placental transporters in health and disease are fairly unknown. To date, P-gp and BCRP have been best characterized in placenta with regards to their dysregulation in the context of underlying maternal diseases and pregnancy complications. However, there are important data on the regulation of some SLCs such as OCT3, OAT4, and OATP2B1 which have potential implications in disease progression and therapeutic intervention. Nucleoside transporters such as ENT1/2 have also been studied because of their impact on the distribution and fetal exposure to antiviral agents. Although crucial information on the possible regulatory mechanisms of these transporters has been obtained through in vitro studies using choriocarcinoma cell lines, primary cultures of syncytiotrophoblasts, or human placental explants, the ultimate proof of functional significance requires in vivo studies. Animal models provide important data on the expression and regulation of drug transporters and their impact in drug distribution; however, due to interspecies differences in placental structure and transporter expression/function, models based on human placental tissues or in vivo human studies are essential to draw final conclusions applicable to clinical practice. A better understanding of the regulation of placental drug transporters in normal and pathological pregnancies will aid in predicting fetal drug exposure and in the development of more targeted medication for mothers.

For both the BBB and BPB, there are still inconsistencies regarding the abundance as well as the cellular and subcellular localization for many transporters. There are also discrepancies between research groups and studies reporting expression or localization of drug transporters in these critical protective barriers. However, in order to properly investigate the net contribution of specific transporters at barriers possessing multiple transport systems, we need information on their abundance and polarized localization as well as function. Considering that most regulatory pathways modify numerous transporters at the same time, investigations would certainly profit from more specific substrates. Moreover, improvements are needed in the analytical or imaging methods to assess the functionality of these transporters in human tissues. If possible, these should be analyses via mathematical modeling of such in vitro data, with the aim to establish in vitro-to-in vivo extrapolation options for a set of compounds, since this will allow integration of such data into existing physiologically based pharmacokinetic/pharmacodynamic models. However, as multicell models are generally required, the complexity to translate such assays to the in vivo situation will be challenging. To date, more information has been generated from placental studies as compared to brain. This is mainly due to the limitations to obtain adequate brain tissue samples from healthy or disease populations. Apart from cultured cell models, most information on the regulation of transporters in human brain comes from postmortem studies or genetic screening of tumor biopsies. More recently, valuable information has also been generated through noninvasive imaging studies. Development of more specific, labeled substrates will improve current knowledge on transporter expression in the brain and will thus allow more in vivo insights into the functional regulation of these transporters.

## 5. Conclusions

While we have identified many gaps in our knowledge, we are beginning to understand the underlying mechanistic pathways and connectivity that regulate transporter expression and activity in critical barriers such as in brain and placenta. Understanding transporter regulation in those barriers will be instrumental in gaining access to new medication for widespread diseases.

## Figures and Tables

**Table 1:** Summary of ABCB1/P-gp alterations in the placenta in different physiological/pathological conditions.

Condition/Factor	Effect			Reference
	mRNA	Protein	Other	
**Based on Clinical Data:**				
Gestational Age	↓ 6.5-fold	↓ 5.1-fold	mRNA + corr with hCG-β	[27]
Gestational Age	-	↓ 69%	-	[26]
SGA (Preterm)	↓	↓	-	[168]
Chorio (Preterm)	↑ (trend)	↔	mRNA + corr with IL-6, IL-1b, TNF-a	[169]
Chorio (Preterm)	↑	-	mRNA + corr with IL-8	[170]
Chorio (Preterm)	↑ 1.61-fold	↔	mRNA + corr with Chorio degree	[171]
IUFGR	↓	-	mRNA + corr with ABCG2 mRNA	[172]
Obesity	↓	↓	-	[173]
Preeclampsia	↔	Ns (↓ trend)	-	[174]
Severe early onset preeclampsia	↔	↓	-	[175]
Diabetes	↑	↔	-	[176]
HIV	↑ 5.5-fold	↑ (trend, *p* = 0.11)	mRNA + corr with estradiol	[177]
Hepatitis	↑ 2.5-fold	↑ 3.1-fold	-	[178]
Maternal betamethasone therapy	↔	-	-	[168]
**Based Placental Explants:**				
LPS	↓ (T1)	↓ (T1)	-	[179]
Poly I:C	↓ (Term)	↔ (Term)	mRNA + corr with TLR3 and TLR4	[179]
Hypoxia	↑ (Term)	↑ (Term)	↓ mRNA of VEGF	[180]
cART treatment	↑1.6- to 2.5-fold	-	-	[177]
**Based Primary Trophoblasts:**				
TNF-a	↓ 45%	↓ 50%	-	[181]
IL-1b	↓ 45%	↓ 50%	-	[181]
Estradiol	↑ ~50%	↑ ~60%	↑ function	[181]
Progesterone	↔	↑ ~40%	-	[181]
cART treatment	-	↑ 2-fold	↑ function	[182]
Prostaglandin E2	↔	↔	↔	[183]

**SGA**: Small for Gestational Age. **hCG-β**: Human Chorionic Gonadotropin-β. **corr**: correlated. **Chorio**: Chorioamnionitis. **IUFGR**: Intrauterine Fetal Growth Restriction. **Ns**: nonsignificant. **LPS**: Lipopolysaccharide. **T1**: First trimester. **Poly I:C**: Polyinosinic:polycytidylic acid. **VEGF**: Vascular Endothelial Growth Factor. **cART**: Combination Antiretroviral Therapy. **↓**, **↑** and **↔** stand for decrease, increase and no change, respectively, relative to control or stated condition.

## Data Availability

Data are available in the cited references.
